# Accuracy-based measures provide a better measure of sequence learning than reaction time-based measures

**DOI:** 10.3389/fpsyg.2015.01158

**Published:** 2015-08-11

**Authors:** Kristi Urry, Nicholas R. Burns, Irina Baetu

**Affiliations:** School of Psychology, University of AdelaideAdelaide, SA, Australia

**Keywords:** serial reaction time task, methodology, implicit learning, sequence learning, reaction time, fluid abilities

## Abstract

The Serial Reaction Time Task (SRTT) was designed to measure motor sequence learning and is widely used in many fields in cognitive science and neuroscience. However, the common performance measures derived from SRTT—reaction time (RT) difference scores—may not provide valid measures of sequence learning. This is because RT-difference scores may be subject to floor effects and otherwise not sufficiently reflective of learning. A ratio RT measure might minimize floor effects. Furthermore, measures derived from predictive accuracy may provide a better assessment of sequence learning. Accordingly, we developed a Predictive Sequence Learning Task (PSLT) in which performance can be assessed via both RT and predictive accuracy. We compared performance of *N* = 99 adults on SRTT and PSLT in a within-subjects design and also measured fluid abilities. The RT-difference scores on both tasks were generally not related to fluid abilities, replicating previous findings. In contrast, a ratio RT measure on SRTT and PSLT and accuracy measures on PSLT were related to fluid abilities. The accuracy measures also indicated an age-related decline in performance on PSLT. The current patterns of results were thus inconsistent across different measures on the same tasks, and we demonstrate that this discrepancy is potentially due to floor effects on the RT difference scores. This may limit the potential of SRTT to measure sequence learning and we argue that PSLT accuracy measures could provide a more accurate reflection of learning ability.

## Introduction

The Serial Reaction Time Task (SRTT) assesses the ability to learn sequences of events, and is used in research across a wide range of fields including motor sequence learning, intelligence, aging, and deficits associated with schizophrenia-spectrum conditions and neurological disorders like Parkinson's Disease. However, there have been some concerns raised on whether SRTT provides a valid measure of motor sequence learning (Salthouse et al., 1999; Kaufman et al., 2010). These concerns have not yet been systematically addressed and SRTT continues to be employed in many studies, the findings of which inform current theories of human cognition. Here, we review some criticisms raised against SRTT and present a modified version of the task that allows derivation of novel performance measures. We then present performance data from both the traditional SRTT and our modified version, investigating the validity of measures derived from both tasks.

**Figure 1:**
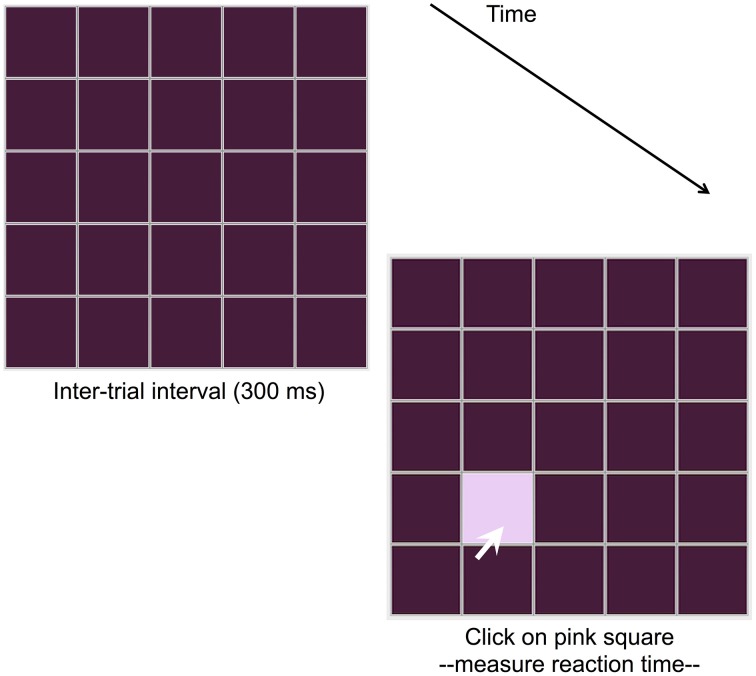
**Serial Reaction Time Task (SRTT) employed in the current study**. Participants see a square illuminate, and must click on it (represented by a white arrow in the figure). In the current study, the delay between a participant response and illumination of the next square is 300 ms.

SRTT is a motor sequence learning task that assesses the acquisition of sequential between-stimuli relations (Nissen and Bullemer, 1987; Robertson, 2007; Rieckmann and Bäckman, 2009). Many variants of the task have been used but they generally share a set of common features: visual stimuli are rapidly presented in a repeating sequence across four possible locations on a computer screen; participants react to each stimulus presentation by pressing the corresponding response key or using a computer mouse to click on the stimulus (see Figure 1); after many presentations of a sequence, blocks of random stimuli are introduced. Sequences can be either non-probabilistic or probabilistic and, importantly, participants are not informed of the sequence. Furthermore, participants are thought to remain unaware of the between-stimuli relations, as revealed by self-report awareness tests, generation tasks, recognition tests, or a combination of these (e.g., Seger, 1994; Reber and Squire, 1994; Rieckmann and Bäckman, 2009).

SRTT is widely used in research on the putative distinction between implicit and explicit cognitive functions. Implicit learning is the incidental acquisition of complex information without awareness of learning or the ability to express learning outside of performance (Seger, 1994; Rieckmann and Bäckman, 2009). Explicit learning is an intentional, re-collective learning process that can be expressed verbally and is thought to be associated with higher-order cognitive functions (Gebauer and Mackintosh, 2007; Kaufman et al., 2010). The distinction between implicit and explicit learning remains one of the most important areas of research in psychology and has informed theories about human cognition, intelligence, and personality (e.g., Estes, 1970; Reber, 1989; Mackintosh, 1998; Lieberman, 2000).

Research has demonstrated patterns consistent with the notion that SRTT is an implicit learning task. First, SRTT performance tends to be unrelated to measures of higher-order cognition (e.g., fluid intelligence: e.g., Rieckmann and Bäckman, 2009; Kaufman et al., 2010) that are generally deemed “explicit” processes (Gebauer and Mackintosh, 2007). This is taken to indicate that SRTT assesses implicit learning because it is assumed that performance on implicit and explicit tasks should be unrelated. There is also some additional evidence that measures of general intelligence correlate with explicit but not incidental conditions on the SRTT (Unsworth and Engle, 2005). Second, performance on SRTT is related to measures of processing speed (PS: e.g., Salthouse et al., 1999; Rieckmann and Bäckman, 2009; Kaufman et al., 2010). PS is considered an evolutionarily older cognitive process (for a more detailed explanation, please see: Reber, 1993) and implicit learning is thought to be a similarly primitive cognitive process (Reber, 1992; Karabanov et al., 2010). Thus, findings that SRTT performance and PS are correlated have been taken to support the notion that SRTT is an implicit learning task.

SRTT performance also appears to be preserved in some populations in which declines on explicit tasks are well documented. For example, while amnesiac patients tend to perform poorly on tasks assessing higher-order cognition because their ability to acquire episodic memories is reduced, in most cases their SRTT performance is preserved (Reber and Squire, 1994; Seger, 1994; Rieckmann and Bäckman, 2009). This suggests that SRTT performance relies on the striatal memory system which is considered implicit, rather than the hippocampal memory system thought to be involved in explicit operations and which is damaged in amnesia (Seger, 1994). Recent neuroimaging studies have supported this hypothesis (Rieckmann and Bäckman, 2009). Similarly, performance on SRTT appears to be largely preserved with age (Rieckmann and Bäckman, 2009), despite a marked age-related decline in fluid abilities (Horn and Noll, 1994) and performance on other learning tasks (Hannah et al., 2012).

Thus, it is inferred that SRTT is an implicit learning task because SRTT performance is unrelated to performance on explicit tasks but is related to measures of PS, and is also preserved in populations in which explicit abilities exhibit decline. However, in the absence of a firm understanding of the cognitive mechanisms underlying implicit vs. explicit processes, such findings are also explained by drawing on the notion that the task is implicit. That is, these results are taken to indicate that the task is an implicit learning task, and these results are also taken to be a consequence of the presumably implicit nature of the task. In this way, the reasoning that SRTT measures implicit learning is rather circular.

Indeed, some methodological concerns have been raised by researchers investigating the nature of sequence learning on SRTT (e.g., Anastasopoulou and Harvey, 1999; Jones and McLaren, 2009). Irrespective of whether the SRTT actually measures implicit learning, an important issue is the fact that the measures derived from the task have been severely criticized (Howard and Howard, 1992; Salthouse et al., 1999; Kaufman et al., 2010). Flaws in the way learning is measured in the task reduce its utility and this motivated our investigation of previously used learning measures and the development of new measures.

SRTT is intended to assess implicit sequence learning via the “efficiency modality” (Seger, 1994), whereby decreases in reaction time (RT) when responding to visual stimuli indicate both the presence and magnitude of sequence learning. There are various methods for quantifying these changes in RT. First, Sequence Learning, or the Trial-Type Effect, is the most accepted and widely used performance measure on SRTT (e.g., Seger, 1994; Salthouse et al., 1999; Robertson, 2007; Siegert et al., 2008; Pederson et al., 2008). It is the difference in RT between random and sequence trial blocks, a random block being a block of trials on which the visual stimuli appear in random order. Second, Total Learning, the reduction in average RT across successive sequence blocks (e.g., Nissen and Bullemer, 1987; Salthouse et al., 1999; Siegert et al., 2008) is commonly used and is assumed to reflect both motor- and sequence-learning because an individual may demonstrate reduced RT across successive sequence blocks due to practice on the task and in the absence of sequence learning (Salthouse et al., 1999; Robertson, 2007; Siegert et al., 2008). It is thus difficult to determine the magnitude of sequence learning from the total reduction in RT over successive blocks of trials, and so Total Learning has commonly been used in conjunction with Sequence Learning. Both measures rely on RT-difference scores and are used to provide a rank ordering of ability to learn on SRTT (Kaufman et al., 2010).

However, some researchers consider RT-difference scores to be too unstable to provide such a rank ordering on learning ability (Howard and Howard, 1992; Kaufman et al., 2010). Consequently, other methods for quantifying learning on the task have been developed. One such measure quantifies learning as relative improvement of RT. That is, the average difference in RT between a random and sequence block is quantified as a ratio (Stevens et al., 2002; Reiss et al., 2006; Pederson et al., 2008, See also: Salthouse et al., 1999). Importantly, whether quantifying sequence learning via raw RT-difference scores or a ratio measure, the variable of interest in SRTT is always RT.

Floor effects may prevent demonstration of learning via RT-difference scores (Salthouse et al., 1999; Kaufman et al., 2010). That is, if an individual is already performing as fast as they physically can, either on the initial sequence blocks or on a random block, then they will be unable to demonstrate sequence learning via a decrease in RT across successive sequence blocks, or on changeover from a random to a sequence block. Consequently, their performance, as measured by RT, will not represent their learning.

Additionally, acquired learning may not necessarily be reflected in RT measures (Kaufman et al., 2010). That is, SRTT only indirectly captures the most important aspect of learning, namely the ability to generate an accurate expectation about the future based on past experience. Inferring the magnitude of learning from RT relies on the assumption that sequence learning allows one to anticipate the next stimulus in the sequence and prepare or initiate the next appropriate movement. But the extent to which the next stimulus is correctly anticipated is either never directly measured because the correct stimulus is shown before the participant makes a response (in SRTT), or only measured during a post-training test intended to assess explicit knowledge of the learned sequence (Nissen and Bullemer, 1987; Seger, 1994; Rieckmann and Bäckman, 2009). This procedure is inconsistent with the way learning is assessed in the vast majority of learning experiments, where the ability to anticipate a future event is typically measured directly. For example, classical conditioning experiments (e.g., Pavlov, 1927) use conditioned responses as indices of learning, whereby learning that a conditioned stimulus (e.g., the sound of a bell) is followed by an unconditioned stimulus (e.g., food) is inferred from anticipatory conditioned responses (e.g., salivation) generated by the presentation of the conditioned stimulus alone. Similarly, in human contingency learning experiments the ability of the participant to anticipate the next correct stimulus is taken as evidence of learning (e.g., Wasserman et al., 1993; Kaufman et al., 2009).

These criticisms of the RT-based measures suggest that the “efficiency modality” may not be the most useful for investigating sequence learning, implicit or otherwise, on SRTT. Alternatively, accuracy of responses could plausibly be more useful for assessing sequence learning because it may reflect the ability to generate the correct sequence. However, accuracy on SRTT is very high and any error is likely to reflect motor rather than predictive error (e.g., magnitude of learning). A task that is identical to SRTT but which assesses sequence learning via the “prediction and control modality” (Seger, 1994)—where participants must predict the location of the next stimulus rather than react to it—may circumvent the problems associated with the “efficiency modality” and RT-based measures. Accordingly, such a task would provide an alternative measure of sequence learning to SRTT.

**Figure 2:**
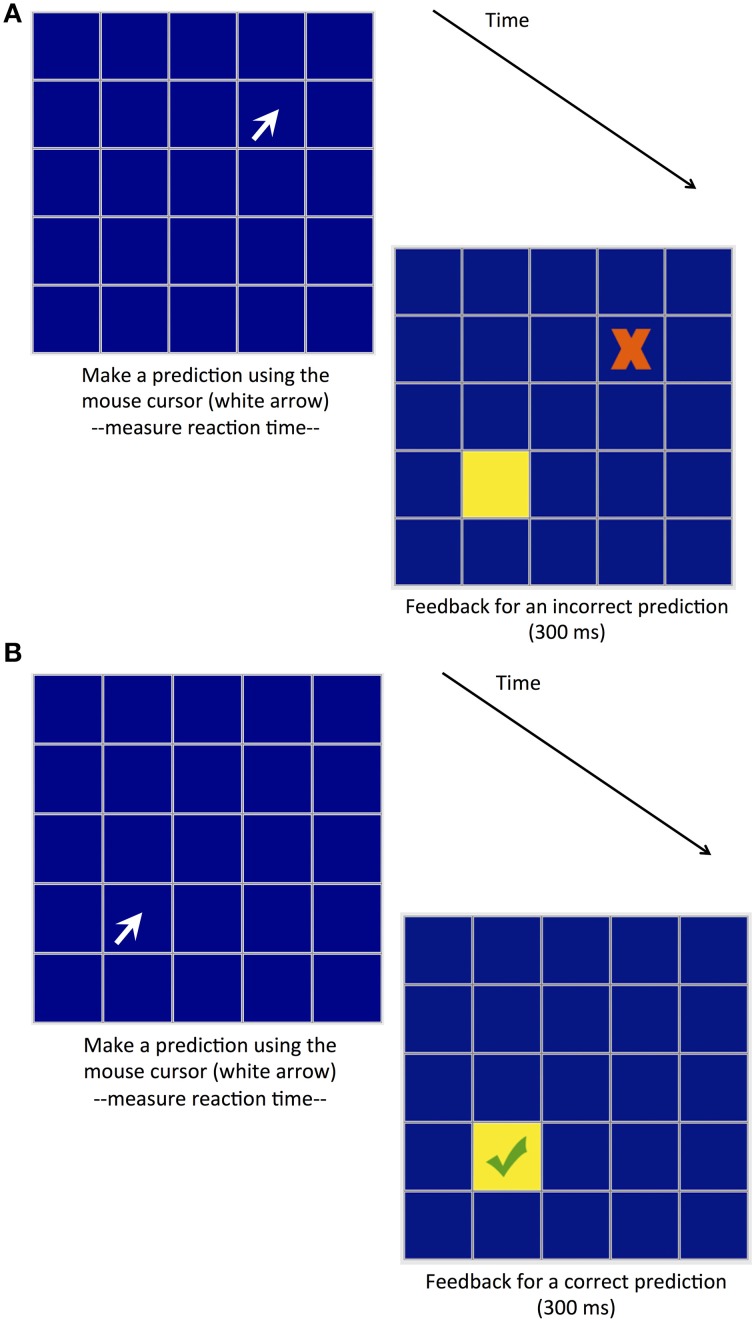
**Predictive Sequence Learning Task (PSLT)**. Participants respond by clicking on a square (represented by a white arrow in the figure), and receive feedback as to the accuracy of their selection: participant responses can be either **(A)** incorrect, or **(B)** correct. Feedback is presented for 300 ms, after which time the participant is required to make another response.

On this basis, we developed a Predictive Sequence Learning Task (PSLT). Like its reactive counterpart, PSLT involves the presentation of a repeating pattern; however, participants are required to predict the location of the next stimulus rather than react to it. That is, in SRTT in the current study a stimulus (a pink square, see Figure 1) is presented at one of four possible locations located within a 5 × 5 grid, and participants are required to react to the stimulus by clicking on it as fast as they can; in PSLT, participants predict the location of the next stimulus (a yellow square, see Figure 2) by clicking on a location of their choice, and feedback on their accuracy is provided so that learning may occur. Participants are not informed of the sequence in either task. Importantly, data on both RT and predictive accuracy for each trial is recorded in PSLT. This means that performance on PSLT can be quantified by RT-based measures, allowing direct comparison with SRTT performance, but also by measures that take into account predictive accuracy.

The current study investigated different methods of quantifying performance on SRTT and PSLT and whether or not performance on these tasks is related to measures of fluid abilities and age. Here, we consider fluid abilities to refer to reasoning ability, working memory, visuo-spatial ability, and PS (e.g., Nettelbeck and Burns, 2010). Our main goal was to investigate the validity of RT-based performance measures for assessing performance on SRTT. Previous findings that SRTT performance does not correlate with fluid abilities (except PS) nor with age may be a result of flawed measurement rather than an actual lack of relations between these abilities, age, and sequence learning. If performance measures that attempt to address and avoid these flaws—i.e., a ratio measure, and performance measured generated on the PSLT—indicate that sequence learning is correlated with fluid abilities and age while traditional measures continue to indicate no relation, then it may indicate that the new measures provide a better measure of sequence learning.

Previous studies have tended to report that SRTT performance is not correlated with higher-order abilities; however, these studies employed only RT-difference measures to quantify SRTT performance. No study has employed a ratio measure to investigate how SRTT performance and fluid abilities are related. Accordingly, potential variation in SRTT performance between RT-difference scores and a ratio measure was investigated here. Similarly, potential variation in PSLT performance between RT-based and accuracy-based measures was also investigated.

Comparison between SRTT and PSLT, that is, between RT-based and accuracy-based performance measures, could also be useful in addressing a theoretical inconsistency regarding SRTT performance and age. SRTT performance is preserved with age and this is cited as evidence for the implicit nature of the task. However, SRTT performance is also positively associated with PS, as discussed above. Given that severe age-related decline in PS is a robust finding (Salthouse, 1996), reports of preserved SRTT performance with age are inconsistent.

We expected to replicate previous findings that SRTT performance is not related to higher-order abilities or age but is positively associated with PS when quantified by RT-difference scores. We also expected this to be true for the RT-difference measures derived from PSLT. As mentioned above, previous findings showed that fluid abilities and age correlate with performance on learning tasks other than the SRTT. Importantly, these other tasks measured learning via predictive accuracy-based measures rather than RT-difference scores, which are prone to floor effects. Both the ratio and accuracy-based measures should, in principle, be less susceptible to floor effects than the RT-difference measures. Hence, we expected that these measures would behave like learning measures derived from tasks other than SRTT and reveal a positive relationship between learning performance and fluid abilities (Kaufman et al., 2010), and a negative relationship between learning performance and age (Hannah et al., 2012).

## Method

### Participants

Participants were *N* = 99 adults (28 men and 71 women) aged between 18 and 60 years (mean age = 25.1; *SD* = 8.88). An additional two participants participated only in the first of two testing sessions and their incomplete data were removed from the dataset. Recruitment was via email from a research participation pool at the University, and via advertisements placed around the University campus and in local businesses. The majority of participants (92 out of 99) had completed, or were currently completing, a university education. Volunteers were paid AUD$50 for their participation.

Participant eligibility was subject to the following criteria: aged between 18 and 60 years; no major medical or psychiatric conditions; no visual disorders; not taking medications that have sedative or stimulant actions; not used any psychoactive or illicit drugs over the past 6 months; not suffering from drug or alcohol dependence; not smoking more than five cigarettes per day.

### Materials

#### Motor sequence learning tasks

The Serial Reaction Time Task (SRTT) and Predictive Sequence Learning Task (PSLT) were matched in appearance and sequences. Both tasks comprised a 5 × 5 grid in which target squares (stimuli) would illuminate, but the color of the grids and illuminations differed between tasks so as to avoid confusion. In each task, stimuli followed one of two non-probabilistic sequences in which the position of an illuminated target square was perfectly predicted by the position of the previously illuminated square. Sequences were four elements long and each block comprised 12-iterations of a sequence, or 48 stimuli in total. Blocks 1-to-6, 8-to-10, and 12 alternated between sequences 1 and 2. That is, Blocks 1, 3, 5, 8, and 10 comprised 12-iterations of sequence 1, and Blocks 2, 4, 6, 9, and 12 comprised 12-iterations of sequence 2. Two sequences were used rather than only one to allow investigation of both initial learning of a sequence (on Block 1) and adaptation following a switch to another sequence (Robertson and Flowers, 1990); however, this aspect is not relevant here. Blocks 7 and 11 were random blocks in which the same four squares were illuminated as in the sequence blocks but the stimulus locations were randomly generated and not predictable. A random block involved repeatedly presenting the four stimulus locations in random order, where the order of the four locations was randomized every four trials (i.e., once all locations had been shown). Like the sequence blocks, each random block consisted of 48 trials.

**Figure 3:**
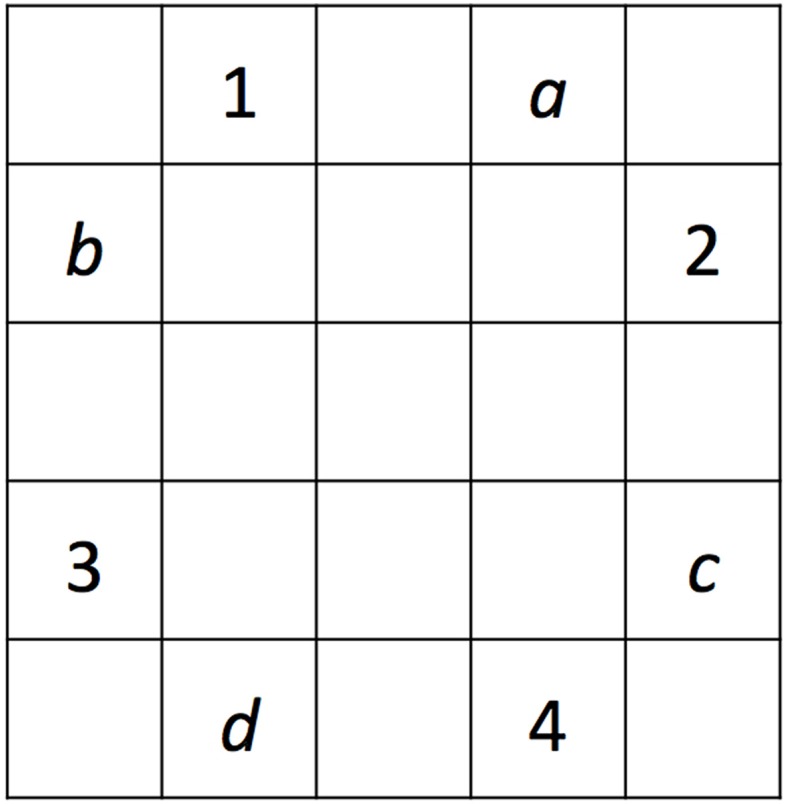
**Illustration of the two pairs of sequences used in PSLT and SRTT**. Numbers 1-4 designate the locations used in one of the two tasks, whereas letters *a-d* designate the locations used in the other task. The two sequences that used locations 1-4 were 3-1-4-2 and 3-2-4-1, and the two sequences that used locations *a-d* were *b-a-d-c* and *b-c-d-a*. The grid locations (1-4 or *a-d*) used in SRTT and PSLT were counterbalanced across participants, as well as which of the two sequences in each set was used as Sequence 1.

The two alternating sequences within each task were designed to be opposite to each other (e.g., used the same four grid locations, or squares) to maximize the amount of interference when they were switched between blocks. The sequences across tasks were designed to be of similar difficulty; however, the grid locations that were used in the two tasks were different to minimize the effect of prior learning on the second learning task (see Figure 3). Participants were not informed of the sequences.

##### Serial reaction time task (SRTT)

SRTT comprised a 5 × 5 grid in which a single square would illuminate at any one time and participants were required to use the mouse to click on the illuminated square as quickly and accurately as possible (see Figure 1). Illumination of a target square ceased on participant response. The inter-trial interval between participant response and the presentation of the next stimulus was 300 ms. If the participant clicked on an incorrect square, a red X appeared inside it and was displayed for the duration of the inter-trial interval. If a correct response was made then only the grid of dark-colored squares was displayed during the inter-trial interval. Both reaction time (RT) and accuracy were measured: RT was the measure of interest for this task and is the response latency between illumination of a target square and participant response; accuracy refers to the distance (in pixels) between the center of the target square and that of the selected square, where a distance of zero indicates a correct response. Although we measured accuracy in SRTT, we expected this measure to show strong floor effects as participants are already shown the stimulus and they are merely asked to click on it. Thus, in SRTT the mean distance between the selected and the correct location should be very close to zero and it is unlikely that this measure will capture individual differences in learning.

##### Predictive sequence learning task (PSLT)

PSLT also comprised a 5 × 5 grid. Participants were required to predict which square would illuminate next by using the mouse to click on the square of their choice (see Figure 2). If their prediction was correct, the square illuminated and a green tick appeared inside the square; if their prediction was incorrect, the correct square illuminated and a red cross appeared inside the selected square. This feedback was presented for 300 ms, after which time it ceased (e.g., all squares turned back to a dark color) and participants were free to make their next selection. This task provided two types of measures—based on predictive accuracy or RT—that may reflect sequence learning. Accuracy refers to the distance (in pixels) between the predicted and actual locations of stimuli, and where a distance of zero indicates a correct response. RT is the length of time it takes a participant to make a response after termination of the feedback from the previous response.

#### Fluid abilities measures

##### Processing speed

Processing speed (PS) was assessed using Inspection Time (Vickers et al., 1972) and the Symbol Digit coding task (McPherson and Burns, 2005). Inspection Time (IT) is the minimum exposure duration required to input critical visual information in order to make a very simple decision with high reliability. IT was estimated from performance on two procedures in which an arrow is presented for increasingly shorter intervals (measured in milliseconds) and then covered by a pattern backward mask. Participants indicate which direction the arrow had been pointing—up or down (vertical procedure), or left or right (horizontal procedure)—by pressing the corresponding key. The scores from these tasks were standardized and averaged for each participant resulting in a single Inspection Time measure.

The Symbol Digit Task is a coding task in which participants must complete as many items as possible in 2 min. A computerized version of the task with mouse response developed by McPherson and Burns (2005) was used in the current study. Each item consists of a symbol presented in the center of the screen, and participants respond by using the mouse to click on a 3 × 3 numerical grid at the bottom of the screen; numbers in the grid correspond to symbols presented in a symbol-number key situated at the top of the screen.

##### Working memory

Working memory was assessed using the Dot Matrix, also known as Spatial Verification Span (Law et al., 1995). This task measures simultaneous storage and processing in the spatial modality. Participants were presented with a blue dot in a 5 × 5 grid and had to remember its location while verifying a matrix addition equation in which two line matrices must be added together to form a third; this was one equation-grid pair. The test has four levels (e.g., 2, 3, 4, and 5 equation–grid pairs; and hence as many dot locations to remember) with four questions per level, for a total for 16 questions. After the level-dependent number of equation-grid pairs had been presented and verified, a blank 5 × 5 grid was presented and participants indicated the location of the previously presented dots by clicking on the corresponding squares in the grid. The number of correct location selections was recorded, and at no point was the task timed. We chose this spatial working memory task (as opposed to verbal alternatives) because spatial ability is relevant to the motor sequence learning tasks in which participants observe sequences of stimuli presented in different spatial locations.

##### Reasoning ability

Reasoning Ability was measured using a computerized short-form version of the Ravens Advanced Progressive Matrices (RAPM) (Raven et al., 2003). Participants identified the missing element that completes a pattern. There were 12-items, and the number of correct items was recorded.

##### Visuo-spatial ability

Visuo-spatial ability is relevant to the motor sequence learning tasks, and was measured using a computerized version of the Mental Rotation Test (Vandenberg and Kuse, 1978). This task assesses the ability to recognize the picture of a 3D shape viewed from different angles: Participants viewed a 3D shape and were required to select the two shapes, from a choice of four, that were images of that shape viewed from different angles. Correct selection of both answers was scored 2, a selection of one and only one correct answer was scored 1, and any other possible answer (e.g., selecting one correct and one incorrect answer) was scored 0. Participants were required to complete 20 items (or as many as possible) in 10 min.

### Procedure

Participants attended two testing sessions that lasted approximately 90 and 40 min, respectively, and that were separated by approximately 1 week. Participants were instructed to not consume any caffeine or other stimulants in the 2 h prior to testing.

In the first testing session, participants commenced the test battery in the order of: an IT task, RAPM (short-form), Dot Matrix task, Symbol Digit Coding Task, the second IT task, an associative learning task, and the Schizotypal Personality Questionnaire (Raine, 1991). The associative learning task and the Schizotypal Personality Questionnaire were used in a related study. The second testing session comprised SRTT, PSLT, and the Mental Rotation Test. The Mental Rotation Test was completed between the SRTT and PSLT, and was followed by a 2-min break. The order of the two IT tasks and the order of the two sequence learning tasks were counterbalanced. Demographic information—age, gender, handedness, and current or completed university degree(s)—was collected at the beginning of the first testing session. Participants were also asked to complete a screening questionnaire investigating medical history and drug use for a related study.

### Ethics statement

This study was approved, and carried out in accordance with recommendations, by University of Adelaide Human Research Ethics Committee. Written informed consent was gained from participants at the start of both testing sessions.

## Results

Descriptive statistics for the Fluid abilities measures and age are provided in Table 1.

**Table 1:** **Intercorrelations between fluid abilities measures and age**.

**Fluid abilities measures and age**	**RAPM**	**Dot matrix**	**Inspection time**	**Symbol digit**	**Mental rotation**	**Age**
RAPM	−					
Dot matrix	^**^0.44	−				
Inspection time	^**^− 0.46	^**^− 0.46	−			
Symbol digit	^**^0.31	0.17	^**^− 0.31	−		
Mental rotation	^**^0.56	^**^0.50	^**^− 0.49	^**^0.40	−	
Age	−0.12	^*^−0.24	^**^0.31	^**^− 0.40	^**^− 0.27	−
Mean	6.65	41.30	0.00	84.10	22.20	25.10
SD	3.21	7.80	0.84	16.20	10.90	8.88

RAPM, Ravens Advanced Progressive Matrices (number of correct items); Dot matrix, Dot Matrix Task (number of correct items completed); Inspection time, averaged z-score from two Inspection Time tasks; Symbol digit, Symbol-Digit Coding Task (number of correct items completed); Mental rotation, Mental Rotation Task (number of correct items). Any absolute r greater than 0.20 is statistically significant at

* p < 0.05, and greater than 0.27 is significant at

** p < 0.001.

Note that smaller Inspection Time scores indicate higher Processing Speed.

### RT-difference scores

**Figure 4:**
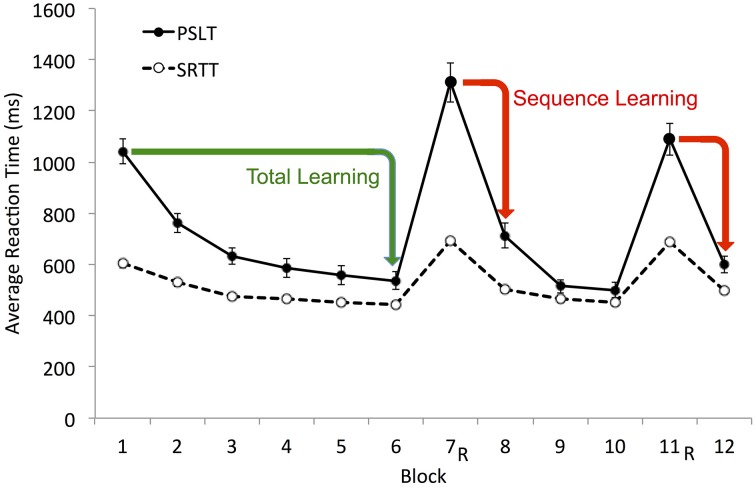
**Illustration of Total and Sequence Learning measures**. Average group data is shown. Total Learning, difference between mean RT on Block 1 and Block 6 (Block 1–Block 6); Sequence Learning, the average drop in mean RT from the two random blocks (7 and 11) to the proceeding sequence blocks (8 and 12, respectively). These RT-difference scores for PSLT are illustrated by the green and red arrows. R, random block; PSLT, Predictive Sequence Learning Task; SRTT, Serial Reaction Time Task. Error bars represent the standard error of the mean. The SRTT data illustrated here includes the RT from all trials; see Supplementary Material Figure 1 for a different version of this figure showing the SRTT data excluding error trials.

Sequence Learning on SRTT has commonly been calculated as the difference in RT on a random block and either the preceding or succeeding sequence block (e.g., Seger, 1994; Salthouse et al., 1999; Robertson, 2007; Pederson et al., 2008). Total Learning on SRTT, assumed to reflect both motor- and sequence- learning, has been inferred from the difference in RT on the first and last sequence blocks (e.g., Nissen and Bullemer, 1987; Salthouse et al., 1999; Siegert et al., 2008). Both measures were calculated in the current study. Sequence Learning was the average drop in mean RT from the two random Blocks (7 and 11) to the subsequent sequence Blocks (8 and 12, respectively; see Figure 4). Total learning was the difference between mean RT on Blocks 1 and 6 (see Figure 4). For this measure, we chose to compare the average RT in Block 1 to that in Block 6 rather than the last Block 12 for two reasons: (i) Block 12 followed a random block and we observed that performance was disrupted in sequence blocks that followed a random block; and (ii) learning had reached asymptote by Block 6.

All trials were included in the RT calculations described above, as well as in the ratio calculation described in the following Section Ratio_−RT_. However, error trials are sometimes omitted from the RT calculations for SRTT, which is inconsistent with our procedure. We chose to include all trials so that we could perform identical RT computations in the two tasks: This is because excluding error trials is a reasonable procedure for SRTT, but not for PSLT, as participants are likely to make many mistakes while they are learning the sequences. Section Reaction Time Data Excluding Error Trials of the Supplementary Material includes all the analyses presented below, but excluding error trials in the RT calculations for SRTT. The SRTT results are very similar if error trials are excluded from the calculations.

All RT performance measures on SRTT and PSLT (Table 2) were significantly greater than zero, indicating that sequence learning did occur [minimum *t*_(98)_ = 9.55, *p* < 0.001].

**Table 2:** **Correlations between RT-based performance measures (Sequence Learning, Total Learning and Ratio_−RT_) on SRTT and PSLT, and fluid abilities measures and age**.

**Fluid abilities measures and age**	**Serial Reaction Time Task (SRTT)**			**Predictive Sequence Learning Task (PSLT)**		
	**Sequence Learning**	**Total Learning**	**Ratio/_−RT_**	**Sequence Learning**	**Total Learning**	**Ratio/_−RT_**
RAPM	^*^ 0.23	0.05	^**^0.33	0.09	−0.04	^**^0.29
Dot matrix	0.19	0.01	^**^0.28	0.17	−0.09	^**^0.41
Inspection time	^*^−0.22	0.02	^**^− 0.32	−0.01	0.11	^*^−0.25
Symbol digit	0.10	−0.02	^**^0.27	−0.13	−0.12	0.19
Mental rotation	^**^0.29	0.03	^**^0.40	0.13	−0.06	^**^0.48
Age	0.09	−0.05	−0.08	^**^0.29	^*^ 0.26	−0.08
Mean	191	160	0.28	469	505	0.41
SD	72.30	122	0.11	488	426	0.22

RAPM, Ravens Advanced Progressive Matrices (number of correct items); Dot matrix, Dot Matrix Task (number of correct items completed); Inspection time, averaged z-score from two Inspection Time tasks; Symbol digit, Symbol-Digit Coding Task (number of correct items completed); Mental rotation, Mental Rotation Task (number of correct items); Sequence Learning, Total Learning, and Ratio_−RT_, performance measures on the motor sequence learning tasks. Any absolute r greater than 0.20 is significant at

* p < 0.05, and greater than 0.27 is significant at

** p < 0.001.

The SRTT data includes the RT from all trials; see Supplementary Material Table 1 for a different version of this table showing the SRTT data excluding error trials.

As expected, Sequence Learning and Total Learning were generally unrelated to measures of fluid abilities on both SRTT and PSLT (see Table 2). Exceptions to this were the weak correlations between SRTT Sequence Learning and performance on the Raven's Advanced Progressive Matrices (*r* = 0.23) and the Mental Rotation Task (*r* = 0.29). This pattern, that SRTT (and PSLT) performance was only weakly correlated with Fluid abilities (only 3 of the 20 relevant correlations reported in Table 2 reached the significance level), replicates the findings of previous studies that have employed these measures.

Sequence Learning and Total Learning on SRTT and PSLT were generally not correlated with measures of PS, with the exception of a weak correlation between SRTT Total Learning and Inspection Time (IT; *r* = 0.22). This lack of a relationship between PS and RT-difference scores was unexpected and does not replicate previous findings.

**Figure 5:**
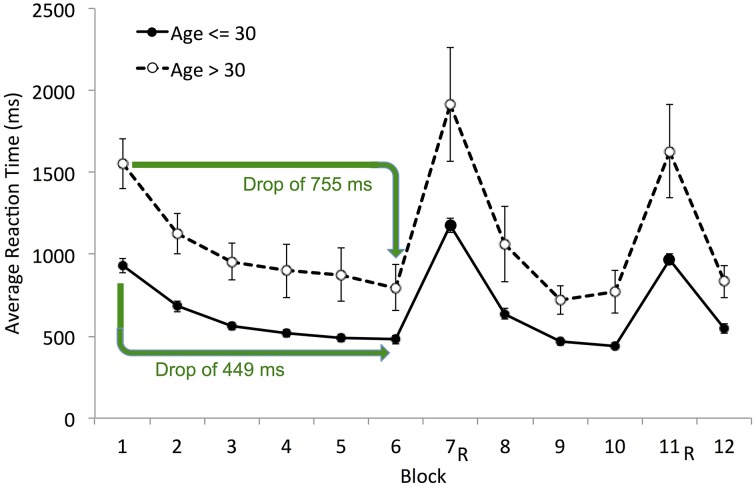
**Mean reaction time (block-by-block) by age group (≤ 30 years and > 30 years) on PSLT**. The green arrows indicate the average drop in RT from Block 1 to Block 6 (i.e., the Total Learning score) for each age group. R, random block. Error bars represent the standard error of the mean.

Sequence Learning and Total Learning were not significantly correlated with age on SRTT and were positively correlated with age on PSLT, suggesting that PSLT performance improved with age. Upon closer inspection, this counterintuitive finding appears to be the result of a floor effect on RT as demonstrated using Total Learning in Figure 5: Younger participants demonstrated faster baseline-RT (e.g., in Block 1) such that they were unable to demonstrate as large an improvement in RT throughout the task as did older, slower participants. Accordingly, older participants had larger Total Learning scores than young participants [*t*_(21.97)_ = 2.46, *p* = 0.022], resulting in the positive age-performance correlation observed. A similar floor effect also occurred for Sequence Learning.

### Ratio_−RT_

A measure of relative improvement in RT, which quantifies learning in the form of a ratio, may minimize floor effects (Stevens et al., 2002; Reiss et al., 2006; Pederson et al., 2008). In the current study, Ratio_−RT_ was calculated for each participant by normalizing the difference in mean RT between random and sequence blocks to the RT for random blocks (Equation 1).

(1) $$\begin{array}{l} \text{Ratio RT}=\frac{\begin{array}{c} \left(\text{R}{\text{T}}_{\text{rand}.\text{block}7}-\text{R}{\text{T}}_{\text{seq}.\text{block}8}\right)\\ +\;\left(\text{R}{\text{T}}_{\text{rand}.\text{block}11}-\text{R}{\text{T}}_{\text{seq}.\text{block}12}\right) \end{array}}{\text{R}{\text{T}}_{\text{rand}.\text{block}7}+\text{R}{\text{T}}_{\text{rand}.\text{block}11}} \end{array}$$

Ratio_−RT_ on both SRTT and PSLT had medium-to-large correlations with performance on all fluid abilities measures (Table 2). This is not consistent with findings using RT-difference measures. Ratio_−RT_ was not associated with age on either task.

### Accuracy-based measures

Learning on PSLT is measured via RT as well as accuracy. Thus, in addition to the RT measures described above, performance on this task was assessed using three accuracy measures:

1. Generation Score refers to the number of trials before participants are able to generate the sequence at least twice, consecutively. Lower scores indicate fewer trials (e.g., predictions) before the correct sequence is generated and hence better performance.
2. Mean Error Score refers to the average distance of participant predictions from the correct sequence. We calculated the distance (in pixels) between the selected grid location and the correct location on every trial, where an accurate prediction results in a distance from the correct location of zero.
3. The Speed-Accuracy Trade-Off Score (Trade-off Score) takes into account both RT and accuracy. It is the multiplicative inverse of the product of the Mean Error Score and mean RT for all predictive blocks. Unlike the two previous measures, higher Trade-Off scores indicate better performance.

Generation Score and Mean Error Score were both positively skewed and so we normalized these measures by taking their inverse but because this did not change the pattern of correlations we present the untransformed data.

Generation Score, Mean Error Score, Trade-off Score had medium-to-large correlations with all fluid abilities measures (Table 3). These results indicate that better reasoning ability, working memory, and visuo-spatial ability, and faster PS were associated with better sequence learning on PSLT.

**Table 3:** **Correlations between accuracy-based performance measures (Generation Score, Mean Error Score, and Speed/Accuracy Trade-off) on SRTT and PSLT, and fluid abilities measures and age**.

**Fluid abilities measures and age**	**Serial Reaction Time Task (SRTT)**		**Predictive Sequence Learning Task (PSLT)**		
	**Mean Error Score**	**Speed/Accuracy Trade-off**	**Generation Score**	**Mean Error Score**	**Speed/Accuracy Trade-off**
RAPM	^*^ 0.21	−0.09	^**^− 0.46	^**^− 0.46	^**^0.45
Dot matrix	0.01	−0.09	^**^− 0.43	^**^− 0.44	^**^0.42
Inspection time	0.05	0.05	^**^0.35	^**^0.33	^**^− 0.49
Symbol digit	^**^0.27	^*^−0.20	^**^− 0.37	^**^− 0.36	^**^0.42
Mental rotation	0.08	^*^−0.23	^**^− 0.60	^**^− 0.59	^**^0.62
Age	−0.16	0.16	^**^0.29	^**^0.27	^**^− 0.29
Mean	8.99	0.45	12.10	66.40	0.05
SD	6.49	0.67	12.90	71.90	0.04

RAPM, Ravens Advanced Progressive Matrices (number of correct items); Dot matrix, Dot Matrix Task (number of correct items completed); Inspection time, averaged z-score from two Inspection Time tasks; Symbol digit, Symbol-Digit Coding Task (number of correct items completed); Mental rotation, Mental Rotation Task (number of correct items); Generation Score, Mean Error Score, and Speed/Accuracy Trade-off, performance measures on the motor sequence learning tasks. A Generation Score cannot be computed for SRTT because participants do not predict the location of the next stimulus as in PSLT. Any absolute r greater than 0.20 is significant at

* p < 0.05, and greater than 0.27 is significant at

** p < 0.001.

**Figure 6:**
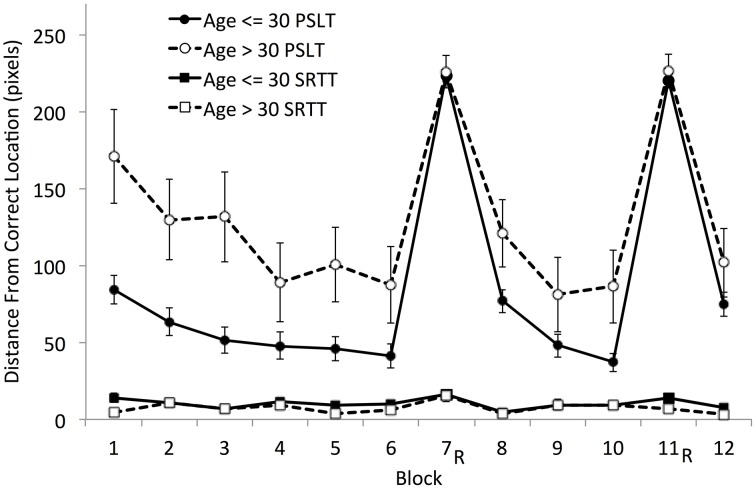
**Mean Accuracy (block-by-block) by age group (≤ 30 years and > 30 years) on SRTT and PSLT**. 100 pixels is the distance between the centers of two adjacent squares. R, random block. Error bars represent the standard error of the mean.

Further, all three accuracy-based measures indicated that PSLT performance declined with age (Table 3). This is also demonstrated in Figure 6 where total Mean Error Score, which was higher for the younger group than the older group [*t*_(20.56)_ = 2.30, *p* = 0.032], was plotted block-by-block for those under- and over- 30 years of age, separately.

We also report the Mean Error Score and the Speed-Accuracy Trade-Off in SRTT (Table 3). As explained previously, we expected strong floor effects in the Mean Error Score that would prevent this measure from being sensitive to individual differences in SRTT; this could also be true of the Speed-Accuracy Trade-Off because the Mean Error Score is used in its computation. As expected, there was a strong floor effect on the Mean Error Scores (the mean was approximately 9 pixels, which represents a very small distance from the correct location as the distance between two adjacent grid locations was 100 pixels). Both the Mean Error Score and the Speed-Accuracy Trade-Off failed to show any meaningful correlations with fluid abilities or age. The correlations were generally weak and failed to reach the significance level, and the few that did suggested that the accuracy measures in SRTT are inversely correlated with fluid abilities (i.e., fluid abilities correlated with poorer performance, i.e., higher Mean Error Scores or a lower Speed-Accuracy Trade-Off).

Overall, these findings suggest that predictive accuracy in PSLT is sensitive to individual differences and shows strong correlations with fluid abilities and an age-related decline in performance. Although accuracy can be measured in SRTT, it does not provide an adequate measure of learning as it suffers from a lack of inter-individual variability and shows an incoherent relationship with fluid abilities.

### Comparison of the RT-difference scores and accuracy-based measures on PSLT

Additionally, we used William's test (Williams, 1959; Steiger, 1980) to investigate if the correlations between PSLT performance and fluid abilities and age were significantly different across performance measures. That is, we tested whether the pattern of correlations with RT-based measures is significantly different from the pattern of correlations with accuracy-based measures. To reduce the number of variables, we calculated z-scores for each type of measure and averaged these to give a single RT-difference score measure and a single Accuracy-based measure of performance for each participant. The single RT measure was achieved by averaging the z-scores for Sequence Learning and Total Learning on PSLT. Ratio_−RT_ was left out because it behaved differently from the other RT-based measures, and was analyzed separately (see the following two Sections). The single Accuracy measure was achieved by averaging the z-scores for Generation Score All Blocks and Speed/Accuracy Trade-off All Blocks. Lower values for the Generation Score indicate better performance, so we computed the additive inverse of the z-scores for this measure before the accuracy-based measure was calculated (so that higher z-scores indicate better performance for both the Generation Score and the Speed/Accuracy Trade-off). Given that the Speed/Accuracy Trade-off is calculated using the Mean Error Score, the latter was excluded from the overall Accuracy measure due to redundancy.

The RT and Accuracy measures were differentially correlated with all fluid abilities and age, as indicated by significant *p*-values for William's test (Table 4). That is, the RT-difference scores and accuracy-based performance measures produced significantly different patterns of correlations within the same task and sample. The accuracy-based measure was significantly correlated with fluid abilities, whereas the RT-difference scores were not. Furthermore, the accuracy-based measure was negatively correlated with age, suggesting an age-related decline in sequence learning. In contrast, the RT-difference scores were positively correlated with age suggesting a counterintuitive increase in sequence learning with age. Thus, opposing conclusions can be drawn depending on the type of measure used to assess sequence learning.

**Table 4:** **Comparison of correlations between RT-difference score and Accuracy-based performance measures on PSLT, and fluid abilities measures and age**.

**Fluid abilities measures**	**Pearson correlation with RT-difference score**	**Pearson correlation with Accuracy-based measure**	***p*-value**
RAPM	0.03	0.50	< 0.001
Dot matrix	0.05	0.47	< 0.001
Inspection time	0.06	−0.46	< 0.001
Symbol digit	−0.16	0.44	< 0.001
Mental rotation	0.04	0.67	< 0.001
Age	0.33	−0.32	< 0.001

RAPM, Ravens Advanced Progressive Matrices (number of correct items); Dot matrix, Dot Matrix Task (number of correct items completed); Inspection time, averaged z-score from two Inspection Time tasks; Symbol digit, Symbol-Digit Coding Task (number of correct items completed); Mental rotation, Mental Rotation Task (number of correct items). p-value, significance value for a William's (1959) test comparing the two correlations between each fluid ability and the two sequence learning measures. Smaller Inspection Time scores indicate faster processing speed; hence, the negative correlation between Inspection Time and the Accuracy measure suggests a positive relationship between processing speed and accuracy.

### Comparison of ratio_−RT_ and the accuracy-based measures on PSLT

Although the RT-difference scores generally failed to show any relationship with fluid abilities and age, Ratio_−RT_ did correlate with fluid abilities. Nevertheless, except for Dot Matrix, Ratio_−RT_ was not correlated with fluid abilities as strongly as the accuracy-based measures (Table 5). Furthermore, the predictive accuracy measures showed an age-related decline in performance that was not detected with Ratio_−RT_. These results suggest that, even though Ratio_−RT_ correlates with fluid abilities to some extent, the accuracy measures are more sensitive to individual differences.

**Table 5:** **Comparison of correlations between Ratio_−RT_ and Accuracy-based performance measures on PSLT, and fluid abilities measures and age**.

**Fluid abilities measures**	**Pearson correlation with Ratio_−RT_**	**Pearson correlation with Accuracy-based measure**	***p*-value**
RAPM	0.29	0.50	0.004
Dot matrix	0.41	0.47	0.411
Inspection time	−0.25	−0.46	0.005
Symbol digit	0.19	0.44	0.001
Mental rotation	0.48	0.67	0.003
Age	−0.08	−0.32	0.002

RAPM, Ravens Advanced Progressive Matrices (number of correct items); Dot matrix, Dot Matrix Task (number of correct items completed); Inspection time, averaged z-score from two Inspection Time tasks; Symbol digit, Symbol-Digit Coding Task (number of correct items completed); Mental rotation, Mental Rotation Task (number of correct items). p-value, significance value for a William's (1959) test comparing the two correlations between each fluid ability and the two sequence learning measures. Smaller Inspection Time scores indicate faster processing speed; hence, the negative correlation between Inspection Time and the Accuracy measure (or Ratio_−RT_) suggests a positive relationship between processing speed and learning.

### Comparison of the ratio_−RT_ and RT-difference scores on PSLT and SRTT

Finally, we compared Ratio_−RT_ with the RT-difference scores because Ratio_−RT_ seemed to provide a more useful measure of learning than the RT-differences scores. Indeed, in general Ratio_−RT_ was more strongly correlated with fluid abilities than the RT-difference scores in both tasks (Tables 6, 7). However, Ratio_−RT_ did not show an age-related decline in performance in either task, although at least it did not correlate positively with age as the RT-difference scores did in PSLT (Table 6). As mentioned previously, this positive correlation between the RT-difference scores and age is most likely due to a floor effect in the younger participants' RT (Figure 5). Thus, neither reaction-time measure demonstrated an age-related decline in performance.

**Table 6:** **Comparison of correlations between Ratio_−RT_ and RT-difference scores on PSLT, and fluid abilities measures and age**.

**Fluid abilities measures**	**Pearson correlation with RT-difference scores**	**Pearson correlation with Ratio_−RT_**	***p*-value**
RAPM	0.03	0.29	0.011
Dot matrix	0.05	0.41	< 0.001
Inspection time	0.06	−0.25	0.002
Symbol digit	−0.16	0.19	0.001
Mental rotation	0.04	0.48	< 0.001
Age	0.33	−0.08	< 0.001

RAPM, Ravens Advanced Progressive Matrices (number of correct items); Dot matrix, Dot Matrix Task (number of correct items completed); Inspection time, averaged z-score from two Inspection Time tasks; Symbol digit, Symbol-Digit Coding Task (number of correct items completed); Mental rotation, Mental Rotation Task (number of correct items). p-value, significance value for a William's (1959) test comparing the two correlations between each fluid ability and the two sequence learning measures. Smaller Inspection Time scores indicate faster processing speed; hence, the negative correlation between Inspection Time and Ratio_−RT_ suggests a positive relationship between processing speed and learning.

**Table 7:** **Comparison of correlations between Ratio_−RT_ and RT-difference scores on SRTT, and fluid abilities measures and age**.

**Fluid abilities measures**	**Pearson correlation with RT-difference scores**	**Pearson correlation with with Ratio_−RT_**	***p*-value**
RAPM	0.20	0.33	0.085
Dot matrix	0.14	0.28	0.067
Inspection time	−0.14	−0.32	0.017
Symbol digit	0.06	0.27	0.006
Mental rotation	0.22	0.40	0.014
Age	0.03	−0.08	0.163

RAPM, Ravens Advanced Progressive Matrices (number of correct items); Dot matrix, Dot Matrix Task (number of correct items completed); Inspection time, averaged z-score from two Inspection Time tasks; Symbol digit, Symbol-Digit Coding Task (number of correct items completed); Mental rotation, Mental Rotation Task (number of correct items). p-value, significance value for a William's (1959) test comparing the two correlations between each fluid ability and the two sequence learning measures. Smaller Inspection Time scores indicate faster processing speed; hence, the negative correlation between Inspection Time and Ratio_−RT_ suggests a positive relationship between processing speed and learning. The SRTT data includes the RT from all trials; see Supplementary Material Table 2 for a different version of this table showing the SRTT data excluding error trials.

## Discussion

The current study investigated the validity of Reaction Time (RT)-based measures commonly used for assessing performance on SRTT. It has been argued that these suffer from floor effects and may otherwise not be reflective of learning (Salthouse et al., 1999; Kaufman et al., 2010); and that performance measures derived from predictive accuracy may provide a more valid assessment. We compared different methods of quantifying performance on SRTT and a novel task that provided meaningful accuracy data—Predictive Sequence Learning Task (PSLT)—within the same sample, exploring how performance on these tasks was related to measures of fluid abilities and age.

The overall pattern of results indicates that, with the exception of age-relations, the two RT-difference scores behaved differently from Ratio_−RT_ and the accuracy-based performance measures on both SRTT and PSLT. The RT-difference scores were generally not associated with fluid abilities on SRTT or PSLT, and suggested no meaningful age-effect on task performance. Ratio_−RT_ was associated with fluid abilities, but not age, on SRTT and PSLT. Similarly, the accuracy-based measures on PSLT were associated with all measures of fluid abilities. The accuracy-based measures also indicated an age-related decline in PSLT performance. The current patterns of results were thus inconsistent across different measures—RT-difference scores vs. Ratio_−RT_ and accuracy-based measures—within the same tasks and sample despite all being assumed to measure the same thing, namely sequence learning. Below we discuss the current findings and their implications in more detail.

### Learning performance and fluid abilities

The current findings were consistent with previous findings that SRTT performance, when quantified by RT-difference scores, is generally not related to reasoning ability, working memory, or visuo-spatial ability (e.g., Rieckmann and Bäckman, 2009; Kaufman et al., 2010). A positive relationship between SRTT performance and processing speed (PS) has been found previously (e.g., Salthouse et al., 1999; Rieckmann and Bäckman, 2009; Kaufman et al., 2010) but was not found here. We suspect that this was because of a PS floor-effect similar to that observed for age. Indeed, a median split on PS revealed a pattern identical to the one illustrated in Figure 5: like the younger group in Figure 5, individuals with faster PS had smaller initial RT, resulting in smaller RT-difference scores compared to those with slower PS. The RT-difference scores produced an identical pattern on PSLT.

Conversely, Ratio_−RT_—a ratio measure that has been employed previously, but not to explore the relationship between SRTT performance and fluid abilities—was associated with reasoning ability, working memory, visuo-spatial ability, and PS on both SRTT and PSLT in the current study.

The three accuracy-based performance measures derived from PSLT were also associated with these fluid abilities. These findings suggest that fluid abilities are associated with sequence learning on SRTT and PSLT, and this is consistent with recent findings that associative learning is related to fluid intelligence (Williams and Pearlberg, 2006; Tamez et al., 2008; Kaufman et al., 2009, 2010).

There is thus a significant discrepancy in the patterns of results produced by different performance measures. Specifically, the RT-difference scores produced a pattern of results expected on an “implicit” learning task, while Ratio_−RT_ and the accuracy-based measures produced patterns of results common to “explicit” learning tasks. Given that this occurred within the same tasks and sample (see Section Comparison of the RT-difference Scores and Accuracy-based Measures on PSLT), the findings support our argument that the RT-difference scores suffer from floor effects that prevent associations between performance and fluid abilities from being detected. Thus, Ratio_−RT_ and accuracy-based measures appear to provide a more valid assessment of performance on SRTT and PSLT.

### Learning performance and age

None of the RT-based measures, including Ratio_−RT_, were meaningfully associated with age on SRTT or PSLT. This is consistent with previous studies, where preservation of SRTT performance with age is cited as evidence for the implicit nature of the task (Rieckmann and Bäckman, 2009).

Conversely, the accuracy-based measures derived from PSLT did indicate an age-related decline in sequence learning. This finding is coherent, given that sequence learning on PSLT was positively related with fluid abilities which also declined with age, and that other forms of learning tend to exhibit age-related decline (Hannah et al., 2012; Tamez et al., 2012). It is also consistent with the cognitive cascade hypothesis of age-related cognitive decline suggested by Tamez et al. (2012), and with some previous research showing an age-related decline in sequence learning (Salthouse et al., 1999; Howard and Howard, 2013).

Howard and Howard (1992) found that age-related decline was especially obvious when participants were required to generate learned sequences, which mirrors our findings with the accuracy-based measures for PSLT. On this basis, it could be argued that the generative nature of PSLT results in the use of a different memory system than that presumably involved in SRTT learning, namely recruitment of the declarative memory system rather than the procedural system, respectively (Roediger, 1990). If this is so, then it is possible that we detected an age-related decline only for the accuracy-based measures in PSLT because older individuals exhibit a decline in declarative memory but not in procedural memory (for a discussion of this hypothesis, see: Howard and Howard, 1992).

However, unlike previous studies, we measured RT and accuracy concurrently (using the PSLT) and hence it is difficult to argue that the performance measures engaged different cognitive mechanisms concurrently within a single task (PSLT) and sample. Differences in the pattern of results produced by RT-based vs. accuracy-based performance measures are thus much more likely to be due to floor effects in RT, as previously discussed. We acknowledge that the age distribution in the current sample was limited (mean age = 25.1; *SD* = 8.88), and replication using an appropriately distributed sample is necessary.

### Limitations

It has not escaped our attention that PSLT is similar to, though is not identical with, the generation tasks often administered to assess explicit knowledge of a sequence learned on SRTT (e.g., Reber and Squire, 1994; Seger, 1994; Pederson et al., 2008; Rieckmann and Bäckman, 2009). On this basis, PSLT may be criticized as being an explicit task and thus not assessing the same type of learning as SRTT. However, the RT-difference scores produced results that mimicked those expected on “implicit” learning tasks. Thus, the PSLT in the current study appears to be measuring both “implicit” and “explicit” learning, depending on which performance measure is being inspected. This clearly demonstrates the circularity of classifying a task as implicit or explicit based on the relationships it shows with other processes that are deemed explicit or implicit. This is not a limitation but, rather, supports our main argument that RT-difference scores are flawed measures.

Another potential limitation is the fact that we used simple, non-probabilistic sequences in SRTT and PSLT. We chose simple sequences because the PSLT is considerably more difficult than SRTT as participants are required to predict the new location rather than simply react to it. Consequently, this limits the complexity of the sequences that can be used since participants may not be able to learn very complex probabilistic or long sequences within a reasonable timeframe. There may be some concern that the current findings are a result of using sequences that were too simple rather than any real difference in the validity of performance measures. However, we replicated previous findings on SRTT (and PSLT) with the RT-difference scores, even though some of these previous studies used probabilistic sequences (e.g., Karabanov et al., 2010; Kaufman et al., 2010), or longer sequences (e.g., Reber and Squire, 1994; Salthouse et al., 1999). It is also worth noting that some studies employing SRTT used simple deterministic sequences similar to ours (e.g., Nissen and Bullemer, 1987; Unsworth and Engle, 2005). Thus, we argue that our findings are relevant to the study of individual differences in sequence learning despite the simplicity of the sequences used, and invite further replication and investigation using more complex sequences.

Finally, another limitation is the possible presence of sequential effects, which may have confounded some of our learning measures. Sequential effects occur when the previous stimulus-response cycles affect the reaction time to a stimulus independently of learning of the sequence. For example, some have reported shorter reaction times when the same stimulus is repeated (e.g., A-A; Kirby, 1976), whereas others have reported shorter reaction times for immediate alternations (e.g., A-B-A; Jarvik, 1951). The reported direction of these effects is not always consistent, and may be influenced by parameters such as the duration of the response-stimulus interval (Soetens et al., 1985). The number of stimuli that intervene between the previous and the current occurrence of a stimulus can influence reaction times even in longer sequences (Hyman, 1953). Anastasopoulou and Harvey (1999) reported that reaction times decrease when more trials intervene between the current and the previous occurrence of the same stimulus. A possible explanation for this effect is that participants might expect that all stimuli will be presented in a minimum number of trials, which would cause them to strongly anticipate a stimulus when its last occurrence happened a relatively long time ago. This sequential effect could have confounded our learning measures that relied on a comparison between sequence and random blocks. This is because during sequence blocks each target location occurred every 4 trials, so participants could have learned this regularity, which might have decreased their reaction times. In contrast, during random blocks each target location occurred on average every 4 trials, but the distance between two trials of the same type varied between 1 and 7 trials (a distance of 1 being an immediate repetition of the same trial type). Because the stimuli were not as predictable during random blocks as they were during sequence blocks, a reaction time difference between the two types of block could be due to a sequential effect instead of, or in addition to, sequence learning.

To investigate this possible sequential effect, we grouped the random block trials depending on the distance from the previous trial of the same type, which was calculated in the same way as Anastasopoulou and Harvey (1999). An analysis comparing reaction times for trials with different distances from the previous trial of the same type is reported in Section Reaction Time Data Controlling for Sequential Effects of the Supplementary Material. As can be seen in Supplementary Figure 2, this distance did influence the reaction times in both tasks.

Because this sequential effect could have confounded some of our sequence learning measures, namely Sequence Learning and Ratio_−RT_ that relied on a comparison between sequence and random blocks, we computed mean reaction times for random blocks controlling for this sequential effect. We did so by using only those trials that had a distance of 4 from the previous trial of the same type, which was the distance for every trial in the sequence blocks. We then computed Sequence Learning and Ratio_−RT_ using these modified reaction times for random blocks. The correlations between these new Sequence Learning and Ratio_−RT_ measures and fluid abilities and age are reported in Supplementary Table 3. These correlations are very similar to the ones reported in Table 2. Thus, this type of sequential effect did not seem to influence our main findings. Nevertheless, future studies could control for sequential effects by using training and pseudorandom sequences with identical transitional properties or subsequences.

## Conclusions

The current findings demonstrate a lack of consistency across performance measures within the same sample and suggest that the RT-difference scores—Sequence Learning and Total Learning—do not provide valid measures of sequence learning on SRTT. This has potentially major implications, given that these measures have been widely used. At best, the findings of previous studies that have employed SRTT must be interpreted critically and with caution. The validity of SRTT itself to assess sequence leaning must be further and critically investigated given that it demands the use of RT-based performance measures. The current findings suggest that, if SRTT were used, a RT ratio measure would be more appropriate for studying individual differences rather than RT-difference scores. Moreover, with further development, PSLT may be a valuable tool for assessing sequence learning either in conjunction with, or in replacement of, SRTT.

### Conflict of interest statement

The authors declare that the research was conducted in the absence of any commercial or financial relationships that could be construed as a potential conflict of interest.

## Abbreviations

PSLT: Predictive Sequence Learning Task (see Section Motor Sequence Learning Tasks)
Ratio_−RT_, A measure of relative improvement in RT: which quantifies learning in the form of a ratio (see Section Ratio_−RT_).
