# Supplementary material for: Accuracy-based measures provide a better measure of sequence learning than reaction time-based measures
Source: Front Psychol. 2015 Aug 11;6:1158. doi: 10.3389/fpsyg.2015.01158 (PMC4531304; doi:10.3389/fpsyg.2015.01158)
Supplement: Supplementary file 1 [file Presentation1.PDF]

Supplementary Material

Accuracy-based measures provide a better measure of sequence learning than reaction time-based measures

Urry[1], K., Burns[1], N. R., & Baetu[1]*, I.

[1] School of Psychology, University of Adelaide, Adelaide, SA 5005, Australia.

*** Correspondence:** Dr Irina Baetu, Level 4 Hughs Building, School of Psychology, University of Adelaide, Adelaide, SA 5005, Australia.
irina.baetu@adelaide.edu.au

1. Reaction Time Data Excluding Error Trials

The following tables and figures show the SRTT data excluding the trials on which participants made an error (i.e., clicked on an incorrect square). These accompany Tables 2 and 7, and Figure 4, from the main text, which show the same data but including all trials in SRTT. As explained in the main text, excluding the error trials in SRTT generates the same pattern of results.

Supplementary Table 1. Correlations between RT-based performance measures (Sequence Learning, Total learning and Ratio$_{-RT}$) on SRTT, and fluid abilities measures and age. RAPM = Ravens Advanced Progressive Matrices (number of correct items); Dot Matrix = Dot Matrix Task (number of correct items completed); Symbol Digit = Symbol-Digit Coding Task (number of correct items completed); Inspection Time = averaged z-score from two Inspection Time tasks; Mental Rotation = Mental Rotation Task (number of correct items); Sequence Learning, Total Learning, and Ratio$_{-RT}$ = performance measures on the motor sequence learning tasks; calculations for these RT measures excluded error trials (unlike Table 2 in the main paper). Any absolute *r* greater than .20 is significant at **p* < .05, and greater than .27 is significant at ***p* < .001.

Fluid abilities measures and age	Sequence Learning	Total Learning	Ratio$_{-RT}$
RAPM	*.29	.05	** .38
Dot Matrix	*.20	.01	** .30
Inspection Time	*-.20	.00	**-.32
Symbol Digit	.15	-.01	** .31
Mental Rotation	**.32	.05	** .44
Age	.06	-.06	-.11
Mean	205	169	.29
SD	73	126	.11

Supplementary Table 2. Comparison of correlations between Ratio$_{-RT}$ and RT-difference scores on SRTT, and fluid abilities measures and age. Smaller Inspection Time scores indicate faster processing speed; hence, the negative correlation between Inspection Time and Ratio$_{-RT}$ suggests a positive relationship between processing speed and learning. RAPM = Ravens Advanced Progressive Matrices (number of correct items); Dot Matrix = Dot Matrix Task (number of correct items completed); Symbol Digit = Symbol-Digit Coding Task (number of correct items completed); Inspection Time = averaged z-score from two Inspection Time tasks; Mental Rotation = Mental Rotation Task (number of correct items). Calculations for these RT measures excluded error trials (unlike Table 7 in the main paper). *p*-value = significance value for a William's (1959) test comparing the two correlations between each fluid ability and the two sequence learning measures.

Fluid abilities measures and age	Pearson correlation with RT-difference scores	Pearson correlation with Ratio$_{-RT}$	*p*-value
RAPM	.24	.38	.677
Dot Matrix	.15	.30	.057
Inspection Time	-.15	-.32	.030
Symbol Digit	.10	.31	.007
Mental Rotation	.27	.44	.023
Age	.00	-.11	.176

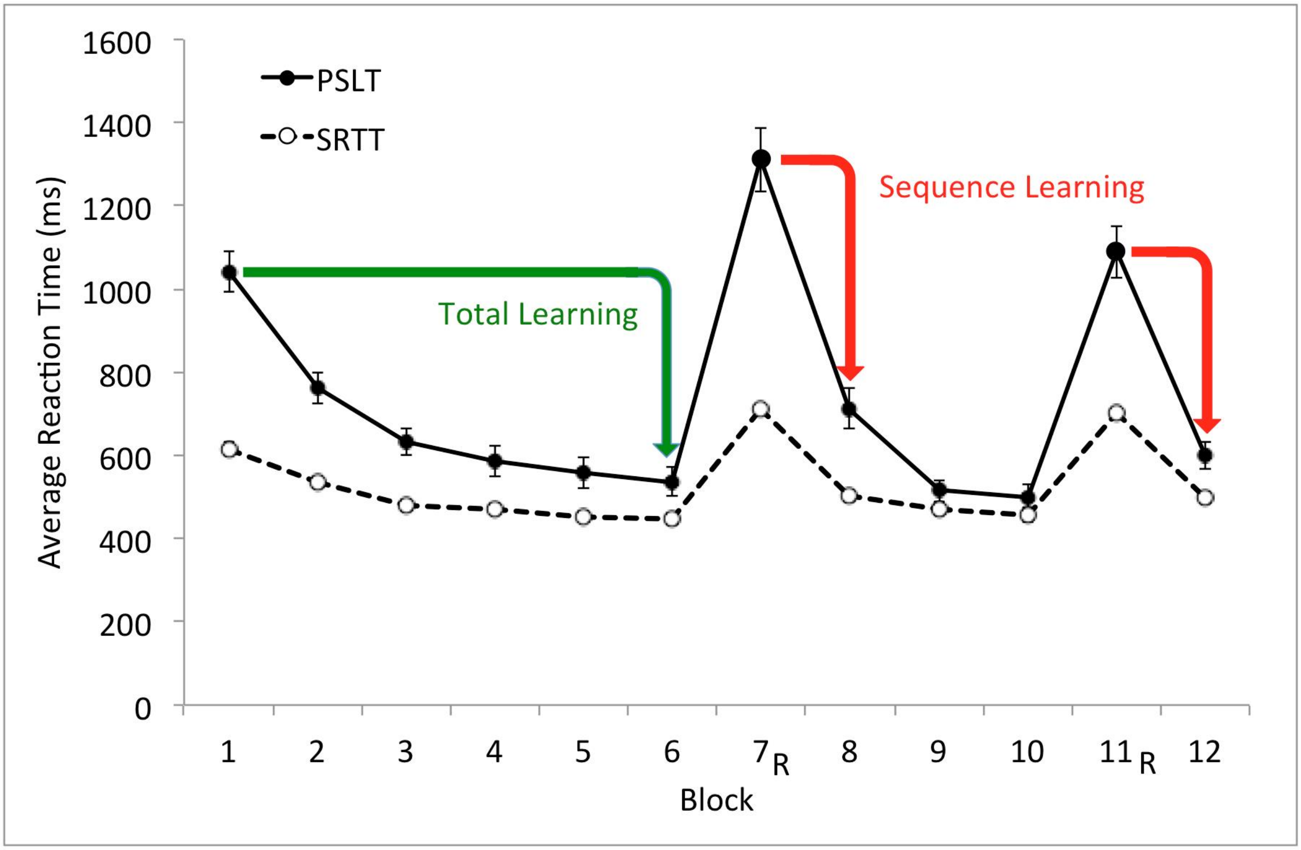

Supplementary Figure 1. Illustration of Total and Sequence Learning measures. Average group data is shown. Total Learning = difference between mean RT on Block 1 and Block 6 (Block 1 – Block 6); Sequence Learning = the average drop in mean RT from the two random blocks (7 and 11) to the proceeding sequence blocks (8 and 12, respectively). These RT-difference scores for PSLT are illustrated by the green and red arrows. R = random block. PSLT = Predictive Sequence Learning Task. SRTT = Serial Reaction Time Task. Error bars represent the standard error of the mean. The SRTT data illustrated here excludes RT data from error trials (unlike Figure 4 in the main paper, which shows RT data from all trials in SRTT).

2. Reaction Time Data Controlling for Sequential Effects

As explained in the main text, a possible sequential effect that could have confounded our learning measures that involved a comparison of sequence and random blocks (i.e., Sequence Learning and $\text{Ratio}_{\text{-RT}}$) is the fact that during sequence blocks each location appeared every 4 trials, whereas this was not the case for the random blocks. In random blocks, the number of intervening trials (i.e. distance) between trials of the same type varied between 1 and 7, where a distance of 1 indicates a repetition (i.e., A-A). Supplementary Figure 3 illustrates the influence of this distance on reaction time for the random block trials. For SRTT, reaction times were longer when the same stimulus location was repeated in fewer than 4 trials (i.e., distances smaller than 4) compared to trials for which the distance from the previous trial of the same type was 4 (minimum $t(98) = 8.24$, $p < .001$). The number of intervening trials had a different effect on the mean reaction times for PSLT. Unlike SRTT, repetitions (i.e., a distance of 1) were associated with faster reaction times compared to trials with a distance of 4 ($t(98) = 3.74$, $p < .001$), whereas alternations (i.e., A-B-A: a distance of 2) were associated with longer reaction times ($t(98) = 1.97$, $p < .052$). We do not report a full set of statistical analyses, suffice it to say that many of these comparisons reach the significance level even after applying a Bonferroni correction for multiple comparisons.

Because this sequential effect affected reaction times in the random blocks, it could have confounded the Sequence Learning and $\text{Ratio}_{\text{-RT}}$ measures that involved comparing sequence and random blocks. We attempted to control for this type of sequential effect by computing the mean reaction times for random blocks using those trials for which the distance is 4, because all sequence block trials had a distance from the previous trial of the same type of 4. We then computed the Sequence Learning and $\text{Ratio}_{\text{-RT}}$ measures using these modified reaction times for the random blocks. The mean Sequence Learning and $\text{Ratio}_{\text{-RT}}$ for each task are reported in Supplementary Table 3, as well a their correlations with fluid abilities and age. Both Sequence Learning and $\text{Ratio}_{\text{-RT}}$ were significantly greater than zero in both tasks, indicating that learning had occurred (minimum $t(98) = 9.67$, $p < .001$). Furthermore, the pattern of correlations with fluid abilities and age is very similar to the one reported in the main text (compare Supplementary Table 3 with Table 2 in the main text).

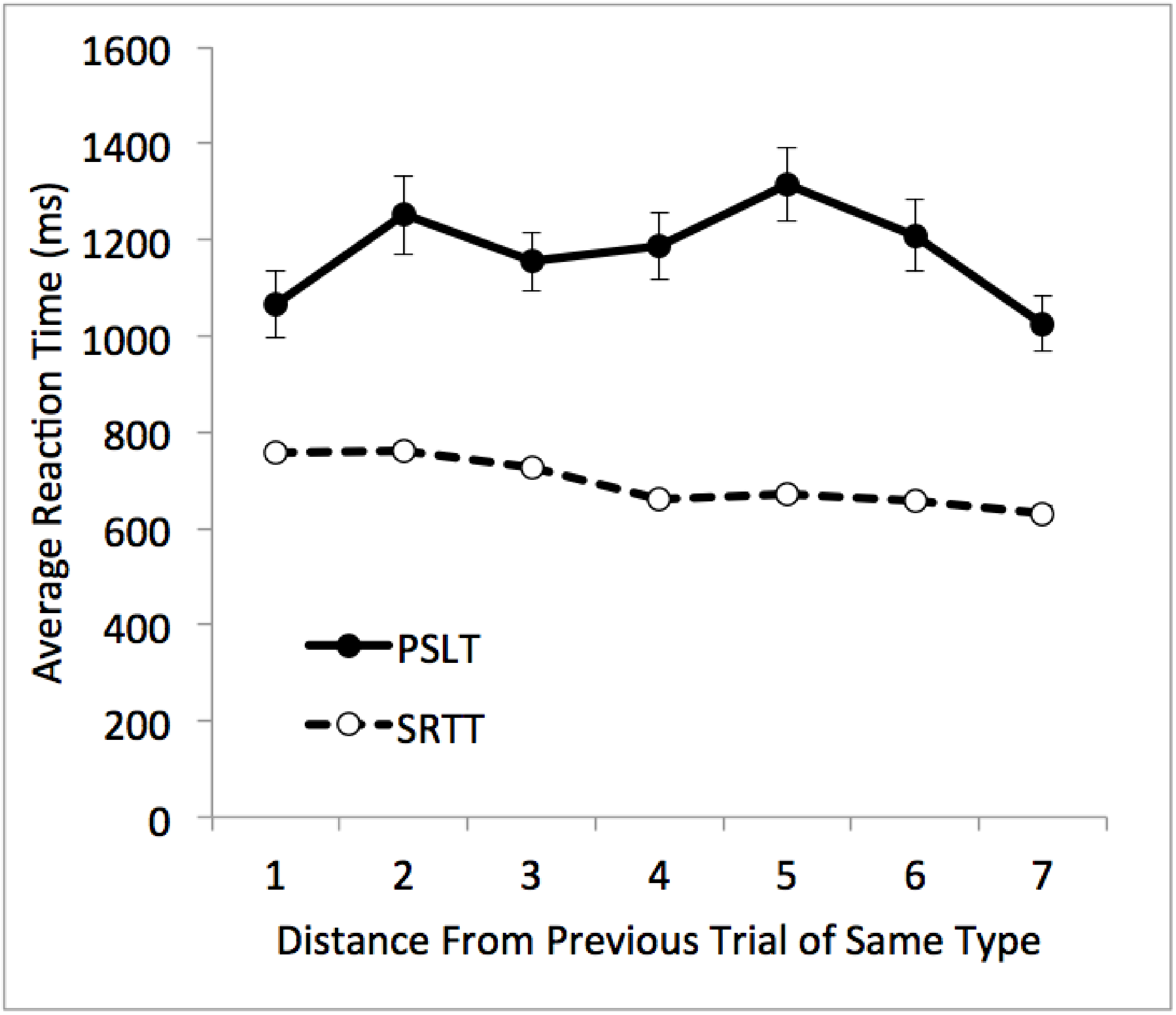

Supplementary Figure 2. Effect of distance from previous trial of the same type on reaction time in random blocks. R = random block. PSLT = Predictive Sequence Learning Task. SRTT = Serial Reaction Time Task. Error bars represent the standard error of the mean. Note that the error bars for SRTT are too small to be visible.

Supplementary Table 3. Correlations between the modified Sequence Learning and Ratio$_{-RT}$ on SRTT and PSLT, and fluid abilities measures and age when trial order sequential effects are controlled for. RAPM = Ravens Advanced Progressive Matrices (number of correct items); Dot Matrix = Dot Matrix Task (number of correct items completed); Symbol Digit = Symbol-Digit Coding Task (number of correct items completed); Inspection Time = averaged z-score from two Inspection Time tasks; Mental Rotation = Mental Rotation Task (number of correct items); Sequence Learning and Ratio$_{-RT}$ = performance measures on the motor sequence learning tasks; calculations for these measures controlled for sequential effects. Any absolute *r* greater than .20 is significant at $*p < .05$, and greater than .27 is significant at $**p < .001$.

	**Serial Reaction Time Task (SRTT)**		**Predictive Sequence Learning Task (PSLT)**	
Fluid abilities measures and age	Sequence Learning	Ratio$_{-RT}$	Sequence Learning	Ratio$_{-RT}$
RAPM	.18	**.28	.12	*.22
Dot Matrix	.12	*.21	.19	**.36
Inspection Time	-.15	*-.26	.00	*-.20
Symbol Digit	.09	*.24	-.11	.17
Mental Rotation	.19	**.31	.15	**.43
Age	.05	-.08	**.32	-.02
Mean	161	.24	533	.39
SD	86	.12	549	.23
